# Bedside quantification of dead-space fraction using routine clinical data in patients with acute lung injury: secondary analysis of two prospective trials

**DOI:** 10.1186/cc9206

**Published:** 2010-07-29

**Authors:** Hassan Siddiki, Marija Kojicic, Guangxi Li, Murat Yilmaz, Taylor B Thompson, Rolf D Hubmayr, Ognjen Gajic

**Affiliations:** 1 Department of Radiology, Mayo Clinic College of Medicine, 200 1stStreet, Rochester 55905, USA; 2 Department of Internal Medicine, Division of Pulmonary and Critical Care Medicine, Mayo Clinic College of Medicine, 200 1stStreet, Rochester 55905, USA; 3 Department of Anesthesiology and Critical Care, Akdeniz University, Dumlupinar Bulvari Kampus, Antalya 0709, Turkey; 4 Department of Medicine, Pulmonary and Critical Care Unit, Medical Intensive Care Unit, Massachusetts General Hospital, Harvard Medical School, 55 Fruit St, Boston, MA 02114, USA

## Abstract

**Introduction:**

Dead-space fraction (Vd/Vt) has been shown to be a powerful predictor of mortality in acute lung injury (ALI) patients. The measurement of Vd/Vt is based on the analysis of expired CO_2 _which is not a part of standard practice thus limiting widespread clinical application of this method. The objective of this study was to determine prognostic value of Vd/Vt estimated from routinely collected pulmonary variables.

**Methods:**

Secondary analysis of the original data from two prospective studies of ALI patients. Estimated Vd/Vt was calculated using the rearranged alveolar gas equation: $\text{Vd}/\text{Vt}=\text{1}-[(0.\text{86}\times {\dot{\text{V}}\text{CO}}_{\text{2est}})/\left(\text{VE}\times {\text{PaCO}}_{\text{2}}\right)]$ where ${\dot{\text{V}}\text{CO}}_{2\text{est}}$ is the estimated CO_2 _production calculated from the Harris Benedict equation, minute ventilation (VE) is obtained from the ventilator rate and expired tidal volume and PaCO_2 _from arterial gas analysis. Logistic regression models were created to determine the prognostic value of estimated Vd/Vt.

**Results:**

One hundred and nine patients in Mayo Clinic validation cohort and 1896 patients in ARDS-net cohort demonstrated an increase in percent mortality for every 10% increase in Vd/Vt in a dose response fashion. After adjustment for non-pulmonary and pulmonary prognostic variables, both day 1 (adjusted odds ratio-OR = 1.07, 95%CI 1.03 to 1.13) and day 3 (OR = 1.12, 95% CI 1.06 to 1.18) estimated dead-space fraction predicted hospital mortality.

**Conclusions:**

Elevated estimated Vd/Vt predicts mortality in ALI patients in a dose response manner. A modified alveolar gas equation may be of clinical value for a rapid bedside estimation of Vd/Vt, utilizing routinely collected clinical data.

## Introduction

Acute lung injury (ALI) and its more severe form acute respiratory distress syndrome (ARDS) are subsets of acute respiratory failure characterized by non-cardiogenic pulmonary edema and severe compromise of gas exchange. The crude incidence of ALI is 78.9 per 100,000 person-years and the age-adjusted incidence is 86.2 per 100,000 person-years. The in-hospital mortality rate of ALI/ARDS remains high despite recent improvements in supportive care [1]. The tools for prediction of prognosis for patients with ALI/ARDS are limited and mostly related to non-pulmonary organ derangements [2-5]. It is surprising that few respiratory variables have shown to predict outcome, as by definition severe respiratory compromise is the main physiological feature in ALI and direct pulmonary insults from pneumonia or aspiration account for more than half of all cases [6,7].

Radiological [8] and histological evidence [9] have shown thrombi in the microvasculature of injured lungs with advanced ALI/ARDS. These thrombi cause ventilation/perfusion (V/Q) mismatch accounting for an increase in physiologic dead space and contribute to elevations in pulmonary vascular resistance [10]. Increased pulmonary dead space fraction (Vd/Vt) proved to be a powerful predictor of mortality in patients with ALI/ARDS enrolled in the trial of low versus high tidal volume [11]. In that trial, Vd/Vt was measured with a bedside metabolic monitor (Deltatrac, Sensor Medics Corp., Yorba Linda, CA, USA), which computes carbon dioxide (CO_2_) production from minute volume and expired gas tensions. As CO_2 _production can also be estimated from the Harris Benedict Equation we reasoned that one might infer Vd/Vt from readily available clinical data [12]. Clinicians at the bedside often calculate partial pressure of arterial oxygen (PaO_2_)/fraction of inspired oxygen (FiO_2_) ratio and alveolar-arterial oxygen gradient to estimate the degree of oxygenation failure. On the other hand the simple calculation of dead space fraction based on minute ventilation (VE), partial pressure of arterial carbon dioxide (PaCO_2_) and estimated metabolic rate (CO_2 _production ${\dot{\text{(V}}\text{CO}}_{2}$)) is seldom used at the bedside. The purpose of this study was to derive and validate a calculation of estimated Vd/Vt as a simple bedside prognostic tool in ALI/ARDS.

## Materials and methods

The institutional review board approved the study protocol and waived the requirements for informed consent for this secondary analysis of two previous prospective studies. ALI and ARDS were defined according to standard American-European consensus conference definitions [13]. Hospital mortality was the primary outcome of this study.

The estimated Vd/Vt was calculated using a rearranged alveolar gas equation for PaCO_2_:

$$PaCO_{2}=\frac{\dot{V}CO_{2}\times 0.863}{VA}$$

where

VA=VE−VD

and VA is alveolar ventilation, VD is dead space ventilation and *0.863 *is a constant necessary for converting fractional concentrations to pressures and correcting volumes to standard conditions [12,14].

$$VD=\text{1}-[(0.\text{86}\times {\dot{\text{V}}\text{CO}}_{\text{2}}{}_{\text{est}})/(\text{VE}\times \text{PaCO2})]$$

where VE is expired minute ventilation and ${\dot{\text{V}}\text{CO}}_{2\text{est}}$ is the estimated production of CO_2 _calculated from the predicted resting energy expenditure equation (REE) [15-18], also known as modified Harris Benedict equation [19].

$${\dot{\text{V}}\text{CO2}}_{\text{est}}=({\text{HB}}_{\text{pred}}\times hf\times 0.\text{8})/\text{6}.\text{8644}$$

where HB_pred _is the predicted REE and is gender specific.

For females = 655.1 + (6.56 × Wt_Kg_) + (1.85 × Ht_cm_) - (4.56 × age)

For males = 66.45 + (13.75 × Wt_Kg_) + (5 × Ht_cm_) - (6.76 × age)

*hf is *hypermetabolic factors:

1.13 per °C over 37°C, 1.2 for minor surgery, 1.35 for major trauma and 1.6 for severe infection [18].

The prognostic value of estimated Vd/Vt was then validated in two recent prospectively collected ALI/ARDS databases, namely Mayo Clinic [20] and ARDS-network [21-23]. Inclusion criteria were patients ventilated for three or more days. The detailed protocols of these original studies have been published previously and a complete description of the methods is available on the internet [24]. Both databases included demographic information (age, height, gender, weight), severity of illness scores (acute physiology and chronic health evaluation (APACHE) III scores and predicted mortality), [24,25] respiratory variables (ventilator tidal volume, minute ventilation, positive end-expiratory pressure (PEEP), peak airway pressure, plateau pressure, FiO_2_, arterial blood gases) collected at the first day of admission (day 1) and day 3, presence of shock (recorded as use of vasopressors), and the duration of mechanical ventilation.

Calculated variables using the above measured parameters included REE, ${\dot{\text{V}}\text{CO}}_{2}$, estimated Vd/Vt, PaO_2_/FiO_2 _ratio, oxygenation index (OI), and quasistatic respiratory compliance (CRS). In the Mayo Clinic validation cohort calculations were performed with and without the correction for hypermetabolic factors. These data were not available in the ARDS-net validation cohort and were not used in final calculations.

### Statistical analysis

Mortality predictions were generated for day 1 and day 3 data. Multiple logistic regression analysis was performed to determine the prognostic value of the estimated Vd/Vt after adjustment for non-pulmonary outcome modifiers. The effect modification by other markers of pulmonary dysfunction (PaO_2_/FiO_2_, OI and CRS) on the association between estimated Vd/Vt and poor prognosis was explored by introducing these variables in the base model. As Vd/Vt may be increased by PEEP-induced overdistension [26] additional adjustment was performed by adding PEEP into the model. Each variable was introduced in the model in units that are clinically intuitive so that the odds ratio and regression estimates generated are simple to interpret. To compare our results with previously published study by Nuckton and colleagues [11], the odds ratio for death was calculated for increments of 0.05 in the Vd/Vt and we used a model consisting of estimated Vd/Vt, CRS and simplified acute physiology score (SAPS II) [27]. However, the latter was only possible in the ARDS-net database because SAPS data were not collected in the Mayo cohort.

All statistical analyses were performed using JMP statistical software (SAS, Cary, NC, USA).

## Results

Variables necessary for calculation of estimated Vd/Vt were recorded in 109 patients in the Mayo cohort and 1,896 patients in ARDS-net cohort (109 patients in the Mayo validation cohort and 1,636 patients in ARDS-net cohort on day 1; and 109 patients in the Mayo validation cohort and 1,395 patients in ARDS-net cohort on day 3). Baseline characteristics of both cohorts are presented in Table 1.

**Table 1:** Baseline characteristics of the two validation cohorts

	Mayo *n *= 109	ARDS-net *n *= 1,896
Age in years, median (IQR)	62 (50-72)	50 (38-64)
Female gender, *n *(%)	56 (51.4)	845 (44.6)
Predicted hospital death, median (IQR)	0.43 (0.19-0.70)	0.31 (0.14-0.58)
PaO2/FiO2 day 1, median (IQR)	118 (82.5-164)	145 (108-195)
PaO2/FiO2 day 3, median (IQR)	175 (117, 241)	155 (114.5-207)
Tidal volume (ml) day 1, median (IQR)	420 (360-500)	420 (350-500)
PEEP (mmH_2_0) day 1, median (IQR)	8 (5-12)	10 (8-14)
Estimated dead-space (%) day 1, median (IQR)	72.5 (64-78.7)	66.3 (57.5-73.6)
Estimated dead-space (%) day 3, median (IQR)	70.8 (61.2-76.3)	68.2 (59.6-75.1)
Hospital mortality *n *(%)	37 (34)	560 (29.5)
Duration of mechanical ventilation median (IQR) (days)	6 (3-11)	10.5 (6-19)

ARDS-net, acute respiratory distress syndrome-network; FiO2, fraction of inspired oxygen; IQR, interquartile range; PaO2, partial pressureof arterial pressure; PEEP, positive end-expiratory pressure.

**Figure 1:**
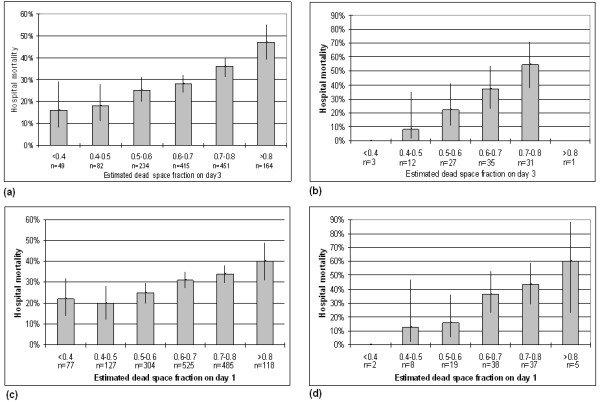
**Univariate analysis of hospital mortality and dead-space fraction**. Shown by increase in percentage mortality for every 10% increase in dead-space fraction **(a) **Day 3 ARDS-net validation cohort (*n *= 1,395). **(b) **Day 3 Mayo Clinic validation (*n *= 109). **(c) **Day 1 ARDS-net validation cohort (*n *= 1,636). **(d) **Day 1 Mayo Clinic validation cohort (*n *= 109). The difference is due to missing data precluding the calculation. Error bars represent 95% confidence intervals. ARDS-net, acute respiratory distress syndrome-network.

The contingency analysis reveals that hospital mortality rises with increasing dead-space percentage (Figures 1a and 1b). This effect was true in both cohorts and held true regardless whether day 1 values were used (Figures 1c and 1d). Both days 1 and 3 estimated Vd/Vt predicted hospital mortality in univariate analysis as well as after adjustment for APACHE III predicted mortality and the presence of shock, and after further adjustment for hypoxemia (PaO_2_/FIO_2 _or OI) and PEEP. The findings were similar in both the Mayo (Table 2) and ARDS-net validation cohorts (Table 3).

**Table 2:** The predictive value of estimated dead-space fraction at day 1 and day 3 of ALI/ARDS in the Mayo validation cohort, outcome hospital mortality

Mortality	Odds ratio	95% CI	
			
*Day 1*			
*(Per 0.05 increment of dead space fraction)*			
**Univariate analysis**			
VdVt	1.33	1.09	1.69
Multivariate analysis			
Base model (Shock + APACHE III predicted mortality), n= 108	1.28	1.04	1.64
Base model + PaO_2_/FiO_2_, n = 108	1.26	1.08	1.61
Base model + OI, n = 107	1.25	1.02	1.61
Base model + PaO_2_/FiO_2 _+ PEEP, n = 108	1.26	1.08	1.64
Base model + PaO_2_/FiO_2 _+ PEEP, n = 107	1.29	1.02	1.69
Base model + Vt, n = 108	1.32	1.05	1.70
*Day 3*			
*(Per 0.05 increment of dead space fraction)*			
**Univariate analysis**			
VdVt	1.47	1.18	1.90
Multivariate analysis			
Base Model (Shock + APACHE III predicted mortality), n = 108	1.43	1.13	1.87
Base model + PaO_2_/FiO_2_, n = 108	1.35	1.05	1.78
Base model + OI, n = 85	1.43	1.03	2.11
Base model + PaO_2_/FiO_2 _+ PEEP, n = 108	1.35	1.05	1.79
Base model + PaO_2_/FiO_2 _+ PEEP, n = 85	1.43	1.03	2.12
Base model + Vt, n = 108	1.47	1.14	1.96

ALI, acute lung injury; APACHE, acute physiology and chronic health evaluation; ARDS, acute respiratory distress syndrome; CI, confidence interval; FiO2, fraction of inspired oxygen; PaO2, partial pressureof arterial pressure; PEEP, positive end-expiratory pressure; OI, oxygenation index; VdVt, dead space fraction; Vt, tidal volume.

**Table 3:** The predictive value of estimated dead-space fraction at day 1 and day 3 of ALI/ARDS in the ARDS-network validation cohort, outcome hospital mortality

Mortality	Odds ratio	95% CI	
			
*Day 1*			
*(Per 0.05 increment of dead space fraction)*			
**Univariate analysis**			
VdVt	1.11	1.06	1.16
Multivariate analysis			
Base Model (Shock + APACHE III predicted mortality), n = 1616	1.09	1.04	1.14
Base model + PaO_2_/FiO_2_, n = 1,610	1.07	1.03	1.13
Base model + OI, n = 1,492	1.08	1.03	1.14
Base model + PaO_2_/FiO_2 _+ PEEP, n = 1,610	1.08	1.03	1.14
Base model + PaO_2_/FiO_2 _+ PEEP, n = 1,492	1.09	1.04	1.15
Base model + Vt, n = 1,616	1.10	1.06	1.16
*Day 3*			
*(Per 0.05 increment of dead space fraction)*			
**Univariate analysis**			
VdVt	1.18	1.12	1.24
Multivariate analysis			
Base Model (Shock + APACHE III predicted mortality), n = 1,369	1.14	1.09	1.21
Base model + PaO_2_/FiO_2_, n = 1,369	1.12	1.06	1.18
Base model + OI, n = 1,241	1.10	1.04	1.17
Base model + PaO_2_/FiO_2 _+ PEEP, n = 1,367	1.10	1.04	1.16
Base model + PaO_2_/FiO_2 _+ PEEP, n = 1,241	1.10	1.04	1.17
Base model + Vt, n = 1,283	1.16	1.10	1.23

ALI, acute lung injury; APACHE, acute physiology and chronic health evaluation; ARDS, acute respiratory distress syndrome; CI, confidence interval; FiO2, fraction of inspired oxygen; PaO2, partial pressureof arterial pressure; PEEP, positive end-expiratory pressure; OI, oxygenation index; VdVt, dead space fraction; Vt, tidal volume.

When the estimated Vd/Vt was adjusted for SAPS II and CRS, the results (odds ratio 1.16, 95% confidence interval (CI) 1.09 to 1.22) were similar to those obtained in the study by Nuckton and colleagues [11].

In the ARDS-net validation cohort, the estimated Vd/Vt on both days 1 and 3 were associated with longer duration of mechanical ventilation in survivors after adjustment for APACHE III predicted mortality, shock, PaO_2_/FIO_2 _and PEEP (mean risk difference of days on mechanical ventilation + 0.3 days, 95% CI 0.1 to 0.5 for day 3; and + 0.2 days, 95% CI 0.03 to 0.4 for day 1). The significance was lost (*P *> 0.05) when PaO2/FiO2 was replaced by OI.

Estimated Vd/Vt correlated weakly with PaO_2_/FiO_2 _(r = -0.30), OI (r = 0.33) and PEEP (r = 0.31).

## Discussion

The results of our study suggest that the estimated Vd/Vt readily calculated from routine clinical data is an independent predictor of hospital mortality in patients with ALI and ARDS. Clinicians at the bedside often calculate PaO_2_/FiO_2 _ratio to estimate the degree of oxygenation failure, although its prognostic value in ALI/ARDS is limited [22,28]. On the other hand, the simple calculation of estimated Vd/Vt, while more informative with regards to degree of pulmonary dysfunction and of higher prognostic value, is seldom used at the bedside.

These results add to the growing evidence that vascular derangement is an important part of ALI phenotype and the level of vascular impairment is a significant predictor of outcome. Previous studies have identified biomarkers of right ventricular dysfunction such as NT-pro brain natriuretic peptide (NT-Pro BNP) as a poor prognostic factor in ARDS patients, probably in the settings of severe pulmonary vascular impairment and right ventricular strain [29].

Our results supplement the findings of Nuckton and colleagues who demonstrated a 45% increased odds of death for every 5% increase in measured Vd/Vt [11]. Lucangelo and colleagues showed that not only the determination of Vd/Vt or capnography derived indices but their evolution during the first 48 hours following intubation could be used in accessing the outcome in ARDS patients [30]. In a recent study by Raurich and colleagues dead space was predictive of mortality during both early and intermediate phase of ARDS [31].

Traditionally, the Enghoff modification of the Bohr equation is used to calculate Vd/Vt and requires a measurement of expired CO_2 _tension by a volumetric capnograph device, thereby limiting its widespread use in clinical practice. Although the measured Vd/Vt has been proven to be a risk factor for both death and prolonged mechanical ventilation in patients with ARDS [10,11,32], our study is the first to show a comparable performance when Vd/Vt is derived from readily available clinical data. The minute ventilation/PaCO_2 _ratio, which is a crude surrogate of the dead-space to tidal volume ratio, was previously reported as an independent risk factor of death in patients with early ALI/ARDS [33]. A related variable, VE40 (defined as hypothetical level of minute ventilation that is required to achieve a 'normal' PaCO_2 _of 40 mmHg) has been used as a weaning index [34] and was independently associated with mortality in a recent Mayo Clinic cohort [20]. This variable, however, does not take into consideration metabolic rate; that is, ${\dot{\text{V}}\text{CO}}_{2}$ was less predictive than estimated Vd/Vt in our study (data not shown) [11].

Enghoff substituted arterial for mean alveolar partial pressure of CO_2 _to derive Vd/Vt. As a result the so-called physiologic dead space is dependent on any mechanism that alters the difference between arterial and mixed expired PCO_2 _[35]. These include ventilation to regions with no blood flow, shunt, V/Q heterogeneity, and oxygen saturation-related changes in the solubility of CO_2 _in blood mediating the Haldane effect. As PEEP influences all four mechanisms, the effects of ventilator management on wasted ventilation as defined by Bohr and physiologic dead-space as defined by Enghoff need not be identical. It is unlikely, however, that this distinction undermines the clinical utility of either surrogate of high V/Q. All clinical estimates of the gas exchange function of the injured lungs are subject to major simplifying assumptions, be they shunt and venous admixture, on the low end of the V/Q spectrum or wasted ventilation and Vd/Vt on the high end of the V/Q spectrum.

Co-morbidities and non-pulmonary organ failures have been shown to carry important prognostic value in patients with ALI/ARDS [3,20]. Previous work has shown an inconsistent relation between a conventional marker of pulmonary organ failure (PaO_2_/FiO_2_) and outcome [22,28], mostly due to its dependence on ventilator settings. OI, on the other hand, takes mean airway pressure into account and may be a more robust marker of pulmonary dysfunction [2,36]. Both measurements depend not only on pulmonary dysfunction but also on changes in cardiac output and oxygen consumption. In our study, estimated Vd/Vt correlated weakly with both PaO_2_/FiO_2 _and OI, and remained independently predictive of poor prognosis.

In recent years there has been an emerging need for a new or expanded definition of ARDS as the definition includes a heterogeneous population, thus creating noise and hampering therapeutic advances in the field. In addition to the proposed level of pulmonary edema [37], a new expanded definition might include a subset of patients with vascular involvement early in the course (based on high Vd/Vt), as those with higher risk of death that could benefit from vascular targeted therapies.

The limitations of our study are related to the observational, secondary analysis design. The presence and timing of measurements were performed according to original study protocol and bedside providers, and not for the purpose of this analysis. In critically ill patients with ALI/ARDS, regional changes in V/Q ratios lead to increases in physiological Vd/Vt. These changes are complex and related not only to vascular obstruction likely to complicate more severe disease but also to alveolar over-distension, such as occurs with high PEEP levels. No data on the use of nitric oxide or prone positioning was available in this study. Of note, introduction of PEEP into our logistic model did not significantly alter the predictive value of estimated Vd/Vt. The 'noise' related to the precision and timing of recording of minute ventilation, PaCO_2 _and the assumptions related to ${\dot{\text{V}}\text{CO}}_{2}$ may have contributed to errors in estimation of Vd/Vt. However, these errors are likely to be evenly distributed between survivors and non-survivors. Perhaps the most noticeable contributor to error would be the absence of point-to-point temporal correlation between arterial blood gas sampling and recording of minute ventilation. Ravenscraft and colleagues [38] have shown that ${\dot{\text{V}}\text{CO}}_{2}$ contributes the least to the excess minute ventilation in patients with ARDS, at least initially. This is likely related to the fact that most patients enrolled in ALI/ARDS datasets are sedated with minimum activity, receive minimal nutrition and are out of the initial shock phase, if present. Another important limitation is that we used the Harris Benedict equation to estimate REE in critically ill patients. The Harris Benedict equation has been developed for healthy subjects, is of limited accuracy in mechanically ventilated patients and inferior to recently validated REE estimation by Faisy and colleagues and Savard and colleagues [16,39]. The comparison of performance of different equations to predict REE was not performed in our study as the pertinent data were not available in both cohorts. Secondly, similarly to the study by Nuckton and colleagues we did not exclude patients with clinical conditions responsible for erroneous values of calorimetric measurements such as hemodynamic and respiratory instability, variations of the CO2 pool, thermogenesis from nutrients and carbohydrate load, airleaks in the respiratory system, accumulation of intermediate metabolites and FiO2 less than 80% [15,16,40]. Many of these conditions are common in the ARDS population at least early in the course of their disease and the utility of findings restricted to patients without hemodynamic and respiratory instability or high levels of FiO2 would be questionable. Even with the limitations of both the simple measurements and the reasonable assumptions, the Vd/Vt estimates performed remarkably well as prognostic factors even though we have not estimated VdVt with the same rigor of prospective trials. This implies that clinicians and clinical epidemiologists can extract useful information about Vd/Vt distributions from relatively simple data. Although estimated Vd/Vt may be of clinical value it still is not equivalent to direct measurements and the use of continuous expired CO_2 _monitoring has the potential advantage of monitoring hemodynamics, patient-ventilator interactions and detection of pulmonary embolism [26].

## Conclusions

Elevated Vd/Vt predicts mortality in ALI patients in a dose-response manner and modified alveolar gas equation allows for its rapid bedside estimation, utilizing routinely collected clinical data. Future studies are needed to validate prognostic value of estimated Vd/Vt in ALI patients and to investigate if specific therapies could improve outcome in patients with elevated Vd/Vt early in the course of the disease.

## Key messages

• Vd/Vt has important prognostic significance in patients with ALI and ARDS, but is not routinely measured in clinical practice.

• In mechanically ventilated patients with ALI and ARDS, Vd/Vt can be estimated from routinely available clinical data (arterial blood gas analysis and minute ventilation).

• Elevated estimated Vd/Vt portends a poor prognosis in patients with ALI and ARDS.

## Abbreviations

ALI: acute lung injury; APACHE III: acute physiology and chronic health evaluation; ARDS: acute respiratory distress syndrome; CI: confidence interval; CRS: quasistatic respiratory compliance; FiO2: fraction of inspired oxygen; OI: oxygenation index; PaCO_2_: partial pressure of carbon dioxide; PaO_2_: partial pressure of oxygen; PEEP: positive end-expiratory pressure; REE: resting energy expenditure equation; SAPS II: simplified acute physiology score; ${\dot{\text{V}}\text{CO}}_{2}$: CO_2 _production; Vd/Vt: dead-space fraction; V/Q: ventilation/perfusion.

## Competing interests

The authors declare that they have no competing interests.

## Authors' contributions

OG designed the research. HS and MY performed data collection and management. HS, MK and GL analyzed the results and drafted the manuscript. OG, RH and TT revised the paper.

## Supplementary Material

Additional file 1 **ARDS-net investigator**. The names and affiliations of ARDS-net investigators. Click here for file

## References

[B1] Rubenfeld GD Caldwell E Peabody E Weaver J Martin DP Neff M Stern EJ Hudson LD Incidence and outcomes of acute lung injury N Engl J Med 2005 353 1685 1693 10.1056/NEJMoa050333 16236739

[B2] Gajic O Afessa B Thompson BT Frutos-Vivar F Malinchoc M Rubenfeld GD Esteban A Anzueto A Hubmayr RD Prediction of death and prolonged mechanical ventilation in acute lung injury Crit Care 2007 11 R53 10.1186/cc5909 17493273 PMC2206401

[B3] Cooke CR Kahn JM Caldwell E Okamoto VN Heckbert SR Hudson LD Rubenfeld GD Predictors of hospital mortality in a population-based cohort of patients with acute lung injury Crit Care Med 2008 36 1412 1420 10.1097/CCM.0b013e318170a375 18434894

[B4] Monchi M Bellenfant F Cariou A Joly LM Thebert D Laurent I Dhainaut JF Brunet F Early predictive factors of survival in the acute respiratory distress syndrome. A multivariate analysis Am J Respir Crit Care Med 1998 158 1076 1081 9769263 10.1164/ajrccm.158.4.9802009

[B5] Luhr OR Antonsen K Karlsson M Aardal S Thorsteinsson A Frostell CG Bonde J Incidence and mortality after acute respiratory failure and acute respiratory distress syndrome in Sweden, Denmark, and Iceland. The ARF Study Group Am J Respir Crit Care Med 1999 159 1849 1861 10351930 10.1164/ajrccm.159.6.9808136

[B6] Vincent JL Akca S De Mendonca A Haji-Michael P Sprung C Moreno R Antonelli M Suter PM The epidemiology of acute respiratory failure in critically ill patients(*) Chest 2002 121 1602 1609 10.1378/chest.121.5.1602 12006450

[B7] Fowler AA Hamman RF Good JT Benson KN Baird M Eberle DJ Petty TL Hyers TM Adult respiratory distress syndrome: risk with common predispositions Ann Intern Med 1983 98 593 597 6846973 10.7326/0003-4819-98-5-593

[B8] Greene R Zapol WM Snider MT Reid L Snow R O'Connell RS Novelline RA Early bedside detection of pulmonary vascular occlusion during acute respiratory failure Am Rev Respir Dis 1981 124 593 601 7305115 10.1164/arrd.1981.124.5.593

[B9] Tomashefski JF Jr Davies P Boggis C Greene R Zapol WM Reid LM The pulmonary vascular lesions of the adult respiratory distress syndrome Am J Pathol 1983 112 112 126 6859225 PMC1916312

[B10] Cepkova M Kapur V Ren X Quinn T Zhuo H Foster E Liu KD Matthay MA Pulmonary dead space fraction and pulmonary artery systolic pressure as early predictors of clinical outcome in acute lung injury Chest 2007 132 836 842 10.1378/chest.07-0409 17573490

[B11] Nuckton TJ Alonso JA Kallet RH Daniel BM Pittet JF Eisner MD Matthay MA Pulmonary dead-space fraction as a risk factor for death in the acute respiratory distress syndrome N Engl J Med 2002 346 1281 1286 10.1056/NEJMoa012835 11973365

[B12] Fenn WO RH Otis AB A theoretical study of the composition of the alveolar air at altitude Am J Physiol 1946 146 637 653 10.1152/ajplegacy.1946.146.5.637 20996488

[B13] Bernard GR Artigas A Brigham KL Carlet J Falke K Hudson L Lamy M Legall JR Morris A Spragg R The American-European Consensus Conference on ARDS. Definitions, mechanisms, relevant outcomes, and clinical trial coordination Am J Respir Crit Care Med 1994 149 818 824 7509706 10.1164/ajrccm.149.3.7509706

[B14] Weir Jd New methods for calculating metabolic rate with special reference to protein metabolism J Physiol 1949 109 1 9 15394301 10.1113/jphysiol.1949.sp004363 PMC1392602

[B15] Weissman Bryan-Brown CW AS Measuring oxygen uptake in the clinical setting Oxygen transport and utilization 1987 Fullerton: Society of Critical Care Medicine p25 64

[B16] Faisy C Guerot E Diehl JL Labrousse J Fagon JY Assessment of resting energy expenditure in mechanically ventilated patients Am J Clin Nutr 2003 78 241 249 12885704 10.1093/ajcn/78.2.241

[B17] Sherman MS A predictive equation for determination of resting energy expenditure in mechanically ventilated patients Chest 1994 105 544 549 10.1378/chest.105.2.544 8306760

[B18] Long CL Schaffel N Geiger JW Schiller WR Blakemore WS Metabolic response to injury and illness: estimation of energy and protein needs from indirect calorimetry and nitrogen balance JPEN J Parenter Enteral Nutr 1979 3 452 456 10.1177/0148607179003006452 575168

[B19] Roza AM Shizgal HM The Harris Benedict equation reevaluated: resting energy requirements and the body cell mass Am J Clin Nutr 1984 40 168 182 6741850 10.1093/ajcn/40.1.168

[B20] Yilmaz M Iscimen R Keegan MT Vlahakis NE Afessa B Hubmayr RD Gajic O Six-month survival of patients with acute lung injury: prospective cohort study Crit Care Med 2007 35 2303 2307 10.1097/01.CCM.0000284505.96481.24 17944018

[B21] Randomized, placebo-controlled trial of lisofylline for early treatment of acute lung injury and acute respiratory distress syndrome Crit Care Med 2002 30 1 6 10.1097/00003246-200201000-00001 11902249

[B22] Ventilation with lower tidal volumes as compared with traditional tidal volumes for acute lung injury and the acute respiratory distress syndrome. The Acute Respiratory Distress Syndrome Network N Engl J Med 2000 342 1301 1308 10.1056/NEJM200005043421801 10793162

[B23] Brower RG Lanken PN MacIntyre N Matthay MA Morris A Ancukiewicz M Schoenfeld D Thompson BT Higher versus lower positive end-expiratory pressures in patients with the acute respiratory distress syndrome N Engl J Med 2004 351 327 336 10.1056/NEJMoa032193 15269312

[B24] Knaus WA Wagner DP Draper EA Zimmerman JE Bergner M Bastos PG Sirio CA Murphy DJ Lotring T Damiano A The APACHE III prognostic system. Risk prediction of hospital mortality for critically ill hospitalized adults Chest 1991 100 1619 1636 10.1378/chest.100.6.1619 1959406

[B25] Teres D Lemeshow S The APACHE III prognostic system Chest 1992 102 1919 1920 10.1378/chest.102.6.1919b 1446536

[B26] Blanch L Romero PV Lucangelo U Volumetric capnography in the mechanically ventilated patient Minerva Anestesiol 2006 72 577 585 16682932

[B27] Le Gall JR Lemeshow S Saulnier F A new Simplified Acute Physiology Score (SAPS II) based on a European/North American multicenter study JAMA 1993 270 2957 2963 10.1001/jama.270.24.2957 8254858

[B28] Bone RC Maunder R Slotman G Silverman H Hyers TM Kerstein MD Ursprung JJ An early test of survival in patients with the adult respiratory distress syndrome. The PaO2/FIo2 ratio and its differential response to conventional therapy. Prostaglandin E1 Study Group Chest 1989 96 849 851 10.1378/chest.96.4.849 2676391

[B29] Bajwa EK Januzzi JL Gong MN Thompson BT Christiani DC Prognostic value of plasma N-terminal probrain natriuretic peptide levels in the acute respiratory distress syndrome Crit Care Med 2008 36 2322 2327 10.1097/CCM.0b013e318181040d 18596623 PMC3106101

[B30] Lucangelo U Bernabe F Vatua S Degrassi G Villagra A Fernandez R Romero PV Saura P Borelli M Blanch L Prognostic value of different dead space indices in mechanically ventilated patients with acute lung injury and ARDS Chest 2008 133 62 71 10.1378/chest.07-0935 17989165

[B31] Raurich JM Vilar M Colomar A Ibanez J Ayestaran I Perez-Barcena J Llompart-Pou JA Prognostic value of the pulmonary dead-space fraction during the early and intermediate phases of acute respiratory distress syndrome Respir Care 55 282 287 20196876

[B32] Kallet RH Alonso JA Pittet JF Matthay MA Prognostic value of the pulmonary dead-space fraction during the first 6 days of acute respiratory distress syndrome Respir Care 2004 49 1008 1014 15329171

[B33] Gattinoni L Vagginelli F Carlesso E Taccone P Conte V Chiumello D Valenza F Caironi P Pesenti A Decrease in PaCO2 with prone position is predictive of improved outcome in acute respiratory distress syndrome Crit Care Med 2003 31 2727 2733 10.1097/01.CCM.0000098032.34052.F9 14668608

[B34] Jabour ER Rabil DM Truwit JD Rochester DF Evaluation of a new weaning index based on ventilatory endurance and the efficiency of gas exchange Am Rev Respir Dis 1991 144 531 537 1892291 10.1164/ajrccm/144.3_Pt_1.531

[B35] Coffey RL Albert RK Robertson HT Mechanisms of physiological dead space response to PEEP after acute oleic acid lung injury J Appl Physiol 1983 55 1550 1557 6358162 10.1152/jappl.1983.55.5.1550

[B36] Seeley E McAuley DF Eisner M Miletin M Matthay MA Kallet RH Predictors of mortality in acute lung injury during the era of lung protective ventilation Thorax 2008 63 994 998 10.1136/thx.2007.093658 18566110 PMC2771451

[B37] Gattinoni L Caironi P Refining ventilatory treatment for acute lung injury and acute respiratory distress syndrome JAMA 2008 299 691 693 10.1001/jama.299.6.691 18270359

[B38] Ravenscraft SA McArthur CD Path MJ Iber C Components of excess ventilation in patients initiated on mechanical ventilation Crit Care Med 1991 19 916 925 10.1097/00003246-199107000-00016 1905215

[B39] Savard JF Faisy C Lerolle N Guerot E Diehl JL Fagon JY Validation of a predictive method for an accurate assessment of resting energy expenditure in medical mechanically ventilated patients Crit Care Med 2008 36 1175 1183 10.1097/CCM.0b013e3181691502 18379244

[B40] Schutz Y The basis of direct and indirect calorimetry and their potentials Diabetes Metab Rev 1995 11 383 408 10.1002/dmr.5610110406 8718497

