# Supplementary material for: Bedside quantification of dead-space fraction using routine clinical data in patients with acute lung injury: secondary analysis of two prospective trials
Source: Crit Care. 2010 Jul 29;14(4):R141. doi: 10.1186/cc9206 (PMC2945122; doi:10.1186/cc9206)
Supplement: Additional file 1 — ARDS-net investigator. The names and affiliations of ARDS-net investigators. [file cc9206-S1.DOC]

**Additional file:**

**Participants in the National Heart, Lung, and Blood Institute (NHLBI) ARDS Clinical Trials Network were as follows**: *Investigators (principal investigators are marked with an asterisk): Cleveland Clinic Foundation* — H.P. Wiedemann,* A.C. Arroliga, C.J. Fisher, Jr., J.J. Komara, Jr., P. Periz-Trepichio; *Denver Health Medical Center* — P.E. Parsons; *Denver Veterans Affairs Medical Center* — C. Welsh; *Duke University Medical Center*— W.J. Fulkerson, Jr.,* N. MacIntyre, L. Mallatratt, M. Sebastian, J. Davies, E. Van Dyne, J. Govert; *Johns Hopkins Bayview Medical Center* — J. Sevransky, S. Murray; *Johns Hopkins Hospital* — R.G. Brower, D. Thompson, H.E. Fessler, S. Murray; *LDS Hospital* — A.H. Morris,* T. Clemmer, R. Davis, J. Orme, Jr., L. Weaver, C. Grissom, F. Thomas, M. Gleich (deceased); *McKay-Dee Hospital* — C. Lawton, J. D'Hulst;*MetroHealth Medical Center of Cleveland* — J.R. Peerless, C. Smith; *San Francisco General Hospital Medical Center* — R. Kallet, J.M. Luce; *Thomas Jefferson University Hospital* — J. Gottlieb, P. Park, A. Girod, L. Yannarell; *University of California, San Francisco* — M.A. Matthay,* M.D. Eisner, J. Luce, B. Daniel, T.J. Nuckton; *University of Colorado Health Sciences Center* — E. Abraham,* F. Piedalue, R. Jagusch, P. Miller, R. McIntyre, K.E. Greene; *University of Maryland*— H.J. Silverman,* C. Shanholtz, W. Corral; *University of Michigan* — G.B. Toews,* D. Arnoldi, R.H. Bartlett, R. Dechert, C. Watts; *University of Pennsylvania* — P.N. Lanken,* J.D. Christie, B. Finkel, B.D. Fuchs, C.W. Hanson, III, P.M. Reilly, M.B. Shapiro; *University of Utah Hospital* — R. Barton, M. Mone; *University of Washington/Harborview Medical Center* — L.D. Hudson,* G. Carter, C.L. Cooper, A. Hiemstra, R.V. Maier, K.P. Steinberg, Margaret Neff, Patricia Berry-Bell; *Utah Valley Regional Medical Center* — T. Hill, P. Thaut; *Vanderbilt University* — A.P. Wheeler,* G. Bernard,*B. Christman, S. Bozeman, T. Swope, L.B. Ware; *Clinical Coordinating Center, Massachusetts General Hospital, Harvard Medical School*— D.A. Schoenfeld,* B.T. Thompson, M. Ancukiewicz, D. Hayden, MA, F. Molay, N. Ringwood, C. Oldmixon, A. Korpak, R. Morse; *NHLBI Staff* — D.B. Gail, A. Harabin,* P. Lew, M. Waclawiw*; *Steering Committee* — G.R. Bernard (chair);*Data and Safety Monitoring Board* — R.G. Spragg (chair), J. Boyett, J. Kelley, K. Leeper, M. Gray Secundy, A.S. Slutsky, B. Turnbull; *Protocol Review Committee* — J.G.N. Garcia (chair), S.S. Emerson, S.K. Pingleton, M.D. Shasby, W.J. Sibbald
